# Call for Papers: Case Reports in Pancreatic Cancer

**DOI:** 10.1089/crpc.2015.29008.cfp

**Published:** 2016-01-01

**Authors:** Charles J. Yeo, Jordan L. Schilling

**Affiliations:** ^1^ Department of Surgery, Thomas Jefferson University, Philadelphia, Pennsylvania.; ^2^ Mary Ann Liebert, Inc., New Rochelle, New York.

Pancreatic cancer poses an enormous challenge to clinicians and cancer scientists because conventional treatments such as surgery and chemotherapy have not been consistently successful. Case reports offer an important opportunity to transfer medical knowledge and stimulate new ideas about clinical care and research direction. More medical reporting is needed to increase the medical knowledge of pancreatic cancer with the aim of leading to significant therapeutic and prognostic progress.

*Case Reports in Pancreatic Cancer *is seeking high quality case reports to publish in future issues. Pertinent topics include:

- New presentations, symptoms, diagnoses, and management of pancreatic cancer.
- Surgical techniques.
- Innovative uses of novel technology, medical devices, or robotics.
- Genetic oncology.
- Chemotherapy and radiation therapy.
- Unusual or unexpected side effects, interactions, or patient response to medication or new technology.
- Unexpected events during patient treatment.
- Family history analysis and epidemiology.
- Interesting findings on the pathogenesis of pancreatic cancer.

Medical reporting and grand rounds are welcome to be submitted as case reports for publication. Please read our *Instructions for Authors* to format your submission: http://www.liebertpub.com/crpc

**Submit online:** https://mc.manuscriptcentral.com/crpc

**Benefits to Authors:**

- High visibility, immediate and unrestricted online access to published articles.
- Rigorous and rapid peer review.
- Easy compliance with open access mandates.
- Authors retain copyright.
- Highly indexed–citation tracking and inclusion in bibliographic databases.
- Targeted email marketing.

**About the Journal:**

*Case Reports in Pancreatic Cancer* is an open access journal publishing authoritative case reports on all aspects of pancreatic cancer diagnosis, management, treatment, and outcomes. The Journal enables physicians, surgeons, oncologists, and the team of professionals that determine and administer care to share their experiences and foster communication and collaboration to optimize patient care.

